# Sequential decarboxylative azide–alkyne cycloaddition and dehydrogenative coupling reactions: one-pot synthesis of polycyclic fused triazoles

**DOI:** 10.3762/bjoc.10.321

**Published:** 2014-12-17

**Authors:** Kuppusamy Bharathimohan, Thanasekaran Ponpandian, A Jafar Ahamed, Nattamai Bhuvanesh

**Affiliations:** 1 PG and Research Department of Chemistry, Jamal Mohamed College, affiliated to the Bharathidasan university, Thiruchirapalli - 620020, Tamilnadu, India; 2 Orchid Chemicals & Pharmaceuticals Ltd, Drug Discovery Research, R&D Center, Sholinganallur, Chennai - 600119, India; 3 Inogent Laboratories Pvt Ltd, API R&D, 28A, IDA, Nacharam, Hyderabad-500076, India; 4 X-ray Diffraction Laboratory, Department of Chemistry, Texas A&M University, College Station, Texas 77842, United States

**Keywords:** copper(II) acetate, decarboxylative CuAAC, dehydrogenative coupling, fused triazoles, one-pot synthesis

## Abstract

Herein, we describe a one-pot protocol for the synthesis of a novel series of polycyclic triazole derivatives. Transition metal-catalyzed decarboxylative CuAAC and dehydrogenative cross coupling reactions are combined in a single flask and achieved good yields of the respective triazoles (up to 97% yield). This methodology is more convenient to produce the complex polycyclic molecules in a simple way.

## Introduction

**Scheme 1:**
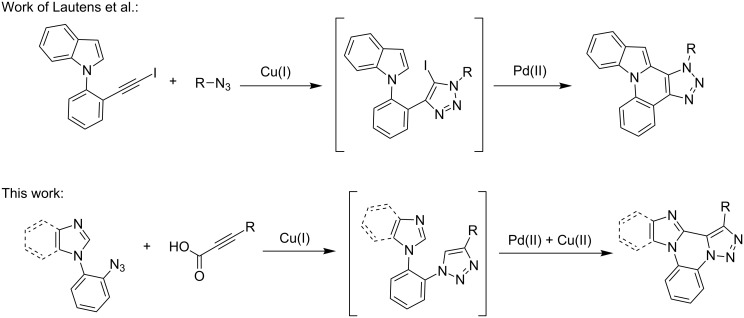
Synthesis of polycyclic fused triazoles.

The copper-catalyzed Huisgen [3 + 2] cycloaddition (or copper-catalyzed azide–alkyne cycloaddition, CuAAC) between an organic azide and a terminal alkyne is a well-established strategy for the construction of 1,4-disubstituted 1,2,3-triazoles [1–4]. In a recent development, this decarboxylative coupling reaction was well documented for the generation of C–C bonds [5]. This method has several advantages over the classical C–C bond formation method including the stability and preparation of the starting material and the non-hazardous byproducts. In 2011, Kolarovič et al. [6] first reported the copper-catalyzed decarboxylative [3 + 2] cycloaddition reaction of 2-alkynoic acid with organic azides. This kind of decarboxylative CuAAC reaction has not been further investigated. Transition metal-mediated C–H bond activation has become a hot topic in recent years [7–11]. Formally, it requires insertion of a transition metal (usually Pd, Ru, Rh or Ir) across a strong C–H bond (90–105 kcal/mol) to form a new, weaker C–M bond (50–80 kcal/mol), followed by generation of a new C–C bond. Generally, transition metal-catalyzed sp^2^ C–H activation is facilitated by directing groups [10–13] or heteroatoms in the heterocyclic compounds [14–18]. This methodology has been applied in the synthesis of polycyclic frameworks as well as in the preparation of biologically important compounds [19–23]. Further development of this reaction has led to double C–H activation which has been used for the construction of biaryl compounds [24–33]. The double C–H activation (dehydrogenative cross coupling) reaction can be classified into two categories: intermolecular and intramolecular. There are several reports in literature describing intermolecular sp^2^ C–H/C–H coupling reactions [24–33], whereas only limited reports are available for intramolecular sp^2^ C–H/C–H coupling reactions [34–38]. Compounds containing a fused triazole skeleton show remarkable biologically activities [39] and new strategies to prepare this class of molecules are highly warranted. Several methodologies were developed for the synthesis of fused triazoles [40]. Ackermann referred to an intramolecular dehydrogenative coupling of 1,4-disubstituted triazoles to achieve tri- and tetracyclic triazoles [34]. Recently, Lautens et al. [41] described a one-pot synthesis of fused triazoles through CuAAC reaction followed by C–H functionalization (Scheme 1).

Specifically, they demonstrated a C–H functionalization of an indole nucleus with 5-iodo-1,2,3-triazoles. In the present study, we replaced the 5-iodo-1,2,3-triazoles with 5*H*-1,2,3-triazoles with intramolecular sp^2^ C–H/C–H cross coupling reaction. To the best of our knowledge, until now there have been no reports describing the combination of decarboxylative CuAAC reaction and C–H activation in an one-pot fashion. This strategy describes the preparation of fused triazoles by one-pot reaction of 2-alkynoic acid and azide derivatives.

## Results and Discussion

According to the report of Kolarovič et al., the decarboxylative CuAAC reaction occurs efficiently with a CuSO_4_/NaAsc/DMSO catalytic system [6]. The palladium-catalyzed oxidative dehydrogenative coupling reaction may be effected by various oxidants [42–43] such as Ag_2_O, AgOAc, Ag_2_CO_3_, Na_2_S_2_O_8_, Cu(OPiv)_2_, Cu(OAc)_2_, benzoquinone and O_2_ among others. In the present study, we have chosen a Cu^2+^ salt because it can be used as an oxidant and as a pre-catalyst for the C–H functionalization and the decarboxylative CuAAC reaction, respectively. 1-(2-Azidophenyl)-1*H*-benzo[*d*]imidazole (**1a**) and phenylpropiolic acid (**2a**) were selected as model substrates to optimize the reaction conditions. Initially, the decarboxylative CuAAC reactions were carried out with 10 mol % of CuSO_4_∙5H_2_O and 20 mol % of NaAsc in DMSO at 80 °C. After 2 h, TLC showed the completion of the cycloaddition reaction and mass spectrometric analysis, [M + 1] peak at 338.1, of the reaction mixture confirmed the formation of **3a**. The reaction mixture was divided into three equal portions and transferred to separate round bottom flasks and the cross coupling was carried out with 5 mol % of three different Pd^2+^ catalysts and 2 equivalents of CuSO_4_∙5H_2_O (Cu^2+^ used for decarboxylative CuAAC) at 120 °C for 12 h. It failed to undergo the oxidative dehydrogenative coupling reaction and the triazole derivative **3a** was isolated in 79–82% yield (Table 1, entries 1–1b). A similar reaction sequence was performed with different copper salts such as CuCl_2_∙H_2_O, Cu(OAc)_2_∙H_2_O and Cu(NO_3_)_2_∙3H_2_O instead of CuSO_4_∙5H_2_O (Table 1, entries 2–4b). Among the Cu^2+^ salts tested, Cu(OAc)_2_∙H_2_O was found to be better than others and yielded 10% of **4a** (Table 1, entries 3–4b). In the literature, we found that additives, such as Brønsted acids, enhance the acidity of the C–H bond in several C–H activation reactions [44–48]. Thus, the reaction was carried out with additives such as pivalic acid, acetic acid or trifluoroacetic acid in the above catalytic system (Table 1, entries 5–5b). When pivalic acid was used, the product formation was improved to 35% (Table 1, entry 5a) whereas acetic acid and trifluoroacetic acid conditions yielded 15% and 19% of **4a**, respectively. None of these modifications provided the desired product in good yield. Therefore, finally we studied the effect of solvents on these reactions. Several polar and non-polar solvents such as dioxane, toluene, 1,2-dichloroethane, DMF, and NMP were tested in this sequential reaction (Table 1, entries 6–10). Toluene was found to be superior to other solvents tested, affording a good yield (87%) of fused triazole **4a** (Table 1, entry 7). No product formation was observed if the reaction was carried out in the absence of Pd(OAc)_2_ (Table 1, entry 11) and without pivalic acid the yield of **4a** was only 22% (Table 1, entry 12). All these results demonstrated that the additive and solvent played a crucial role in the dehydrogenative coupling reaction.

**Table 1:** Optimization of reaction conditions for the preparation of **4a**.

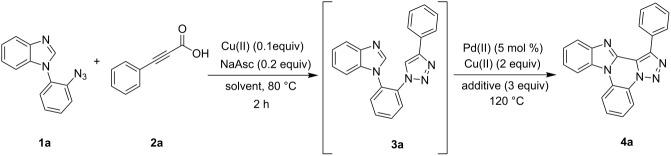							

Entry	Cu^2+^	Solvent	Pd^2+^	Additive	Time [h]	Yield(%)^a^	
						**3a**	**4a**

1	CuSO_4_∙5H_2_O	DMSO	Pd(OAc)_2_	–	12	80	–
1a			PdCl_2_	–		82	–
1b			Pd(PPh_3_)_2_Cl_2_	**–**		79	**–**
2	CuCl_2_∙H_2_O	DMSO	Pd(OAc)_2_	–	12	81	–
2a			PdCl_2_	–		85	–
2b			Pd(PPh_3_)_2_Cl_2_	–		80	–
3	Cu(OAc)_2_∙H_2_O	DMSO	Pd(OAc)_2_	–	12	76	10
3a			PdCl_2_	–		77	trace
3b			Pd(PPh_3_)_2_Cl_2_	–		78	–
4	Cu(NO_3_)_2_∙3H_2_O	DMSO	Pd(OAc)_2_	–	12	50	–
4a			PdCl_2_	–		58	–
4b			Pd(PPh_3_)_2_Cl_2_	–		52	–
5	Cu(OAc)_2_∙H_2_O	DMSO	Pd(OAc)_2_	pivalic acid	12	58	35
5a				AcOH		78	15
5b				TFA		76	19
6	Cu(OAc)_2_∙H_2_O	dioxane	Pd(OAc)_2_	pivalic acid	12	38	39
7	Cu(OAc)_2_∙H_2_O	toluene	Pd(OAc)_2_	pivalic acid	3	–	87
8^b^	Cu(OAc)_2_∙H_2_O	1,2-DCE	Pd(OAc)_2_	pivalic acid	12	46	trace
9	Cu(OAc)_2_∙H_2_O	DMF	Pd(OAc)_2_	pivalic acid	12	70	21
10	Cu(OAc)_2_∙H_2_O	NMP	Pd(OAc)_2_	pivalic acid	12	66	24
11	Cu(OAc)_2_∙H_2_O	toluene	–	pivalic acid	12	97	–
12	Cu(OAc)_2_∙H_2_O	toluene	Pd(OAc)_2_	–	12	75	22

^a^Isolated yield. ^b^Reaction was performed at 100 °C.

**Scheme 2:**
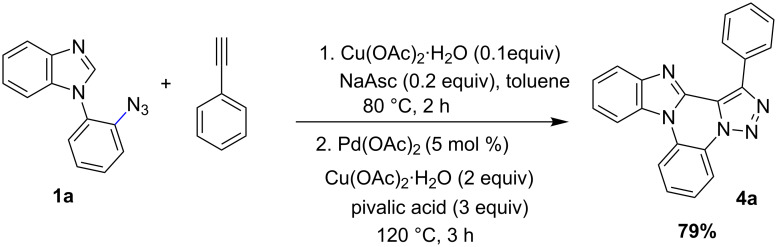
Synthesis of fused triazole **4a** using phenylacetylene.

The sequential reaction was performed with phenylacetylene instead of phenylpropiolic acid and the product **4a** was isolated in 79% yield (Scheme 2). This result clearly shows that the use of 2-alkynoic acid is more advantageous for this reaction.

**Scheme 3:**
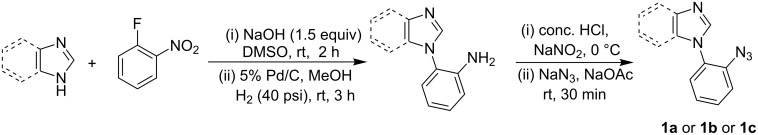
Synthesis of **1a**, **1b** and **1c**.

**Scheme 4:**
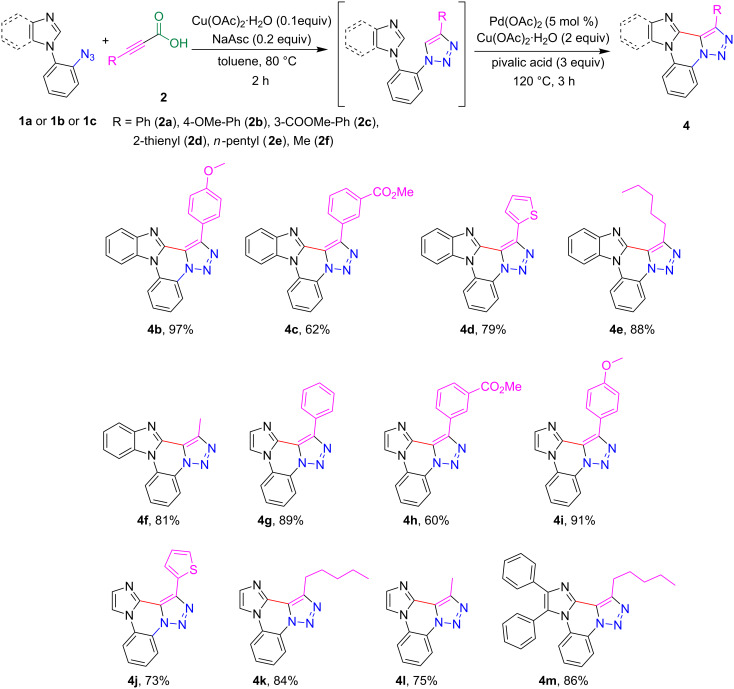
Synthesis of fused polycyclic triazole analogs **4**.

The 1-(2-azidophenyl)-1*H*-imidazole derivatives **1b** and **1c** also participates effectively in the optimized reaction conditions. The azide derivatives **1a, 1b** and **1c** were prepared from 1-fluoro-2-nitrobenzene (Scheme 3) according to literature procedure [49]. Using the optimized reaction conditions, the reactivity of different 2-alkynoic acids was investigated with **1a** and **1b** and the results are shown in Scheme 4.

The electron-donating substituents (OMe)-bearing phenyl ring in **2b** resulted in a good yield of triazole analogs **4b** and **4i** in contrast **2c** with electron withdrawing methoxycarbonyl substitution provided moderate yields of **4c** and **4h** (62% and 60%). Notably, the carboxylate group in **4c** and **4h** offers a versatile synthetic functionality for further derivatization reactions. The thiophene-derived alkynoic acid, **2d**, provided the corresponding triazole analogs **4d** and **4j** in 79% and 73%, respectively. Likewise, the alkynoic acid derived from short and long linear alkyl chains also provided good yields (75–88%) of polycyclic fused triazoles **4e**, **4f**, **4k**, **4l** and **4m** (Scheme 4).

**Figure 1:**
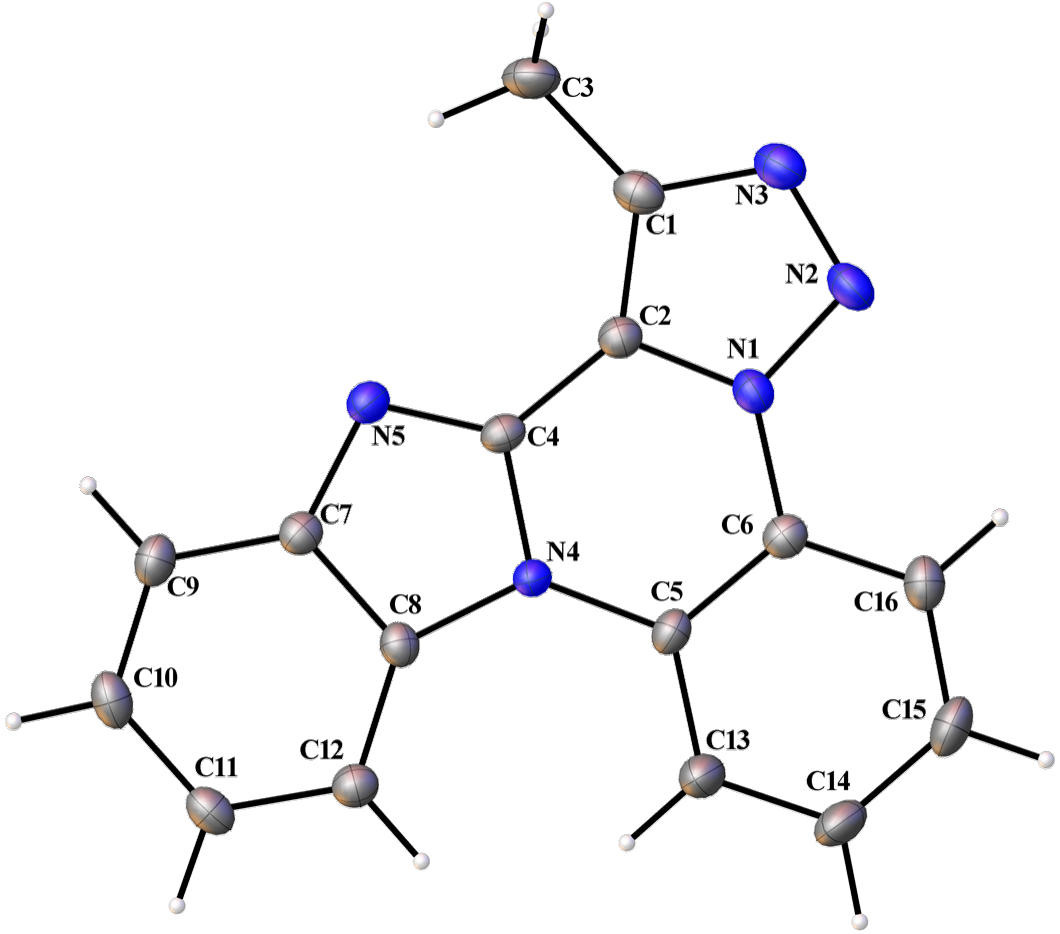
ORTEP diagram of **4f** (CCDC 979471).

The structures were fully characterized by NMR, IR and mass spectroscopic techniques. Furthermore, the structure of **4f** has been confirmed by single crystal X-ray crystallographic study (Figure 1).

**Scheme 5:**
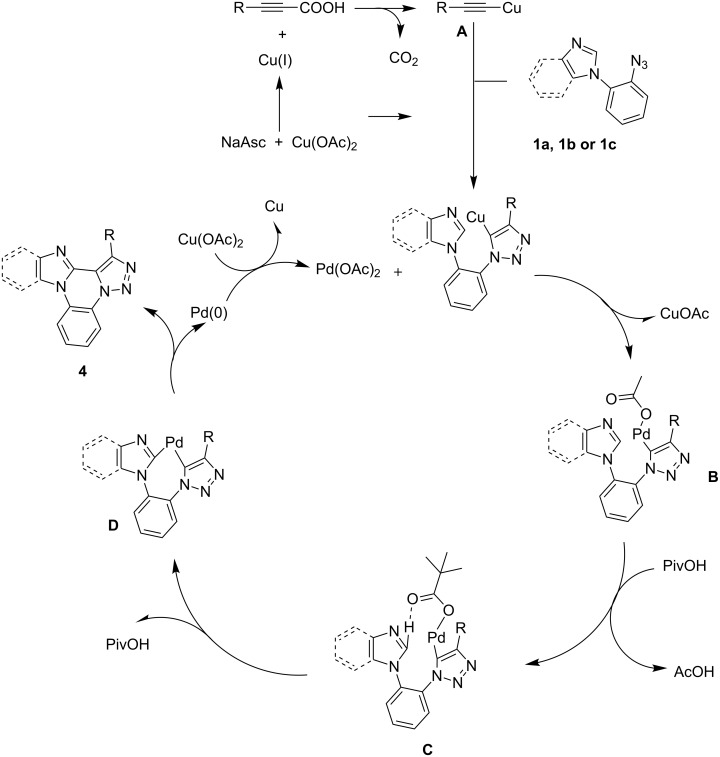
Proposed mechanism for the formation of **4**.

The proposed reaction mechanism for the formation of **4** is described in Scheme 5. Initially alkynoic acid **2** undergoes decarboxylation to form the copper acetylide (**A**) in the presence of the Cu^+^ catalyst which is generated by the reduction of Cu^2+^ with sodium ascorbate. The obtained copper acetylide undergoes regioselective [3 + 2] cycloaddition with azide derivative **1** to yield the copper salt of **3** and a transmetalation reaction gave the intermediate **B**. We assumed that the pivalate group replaces the acetate group in **B** and may produce **C**. The pivalate group in **C** facilitates the palladium insertion to the C–H bond to give **D** and subsequent reductive elimination reaction yields the polycyclic triazoles **4**.

## Conclusion

In summary, we have successfully developed an efficient and convenient one-pot protocol for the synthesis of novel benzimidazole and imidazole-fused 1,2,3-triazoloquinoxaline derivatives. The key finding of this work is the bifunctional behavior of Cu(OAc)_2_∙H_2_O in the reaction sequence.

## Experimental

### General procedure for the synthesis of fused triazoloquinoxaline derivatives **4**

Substituted phenylpropiolic acids (**2**) were prepared by the literature procedure [50]. To a mixture of 1-(2-azidophenyl)-1*H*-benzo[*d*]imidazole (**1a**) or 1-(2-azidophenyl)-1*H*-imidazole (**1b**) (0.85 mmol), 2-alkynoic acid (**2**) (1.02 mmol) and Cu(OAc)_2_∙H_2_O (0.085 mmol, 10 mol %) in toluene (8 mL) was added to sodium ascorbate (0.17 mmol, 20 mol %) at room temperature. The mixture was stirred at 80 °C for 2 h. Cu(OAc)_2_∙H_2_O (1.7 mmol), Pd(OAc)_2_ (0.043 mmol, 5 mol %) and pivalic acid (2.55 mmol) were added into the above reaction mixture and then refluxed at 120 °C for 3 h. The reaction mixture was cooled to room temperature and diluted with ethyl acetate (200 mL). The mixture was filtered through a pad of celite and the filtrate was washed with water, dried over anhydrous Na_2_SO_4_ and concentrated under vacuum. The residue was purified by column chromatography using hexane/ethyl acetate as eluent to obtain the desired product **4** (60–97%).

## Supporting Information

File 1 X-ray crystallographic data of **4f**, characterization, ^1^H and ^13^C NMR data of compounds **3a** and **4a**–**m**.

## References

[1] Rostovtsev V V, Green L G, Fokin V V, Sharpless K B (2002). Angew Chem, Int Ed.

[2] Himo F, Lovell T, Hilgraf R, Rostovtsev V V, Noodleman L, Sharpless K B, Fokin V V (2005). J Am Chem Soc.

[3] Shao C, Wang X, Xu J, Zhao J, Zhang Q, Hu Y (2010). J Org Chem.

[4] Shin J-A, Lim Y-G, Lee K-H (2012). J Org Chem.

[5] Rodriquez N, Goossen L J (2011). Chem Soc Rev.

[6] Kolarovič A, Schnürch M, Mihovilovic M D (2011). J Org Chem.

[7] Dyker G (1999). Angew Chem, Int Ed.

[8] Kakiuchi F, Chatani N (2003). Adv Synth Catal.

[9] Dick A R, Sanford M S (2006). Tetrahedron.

[10] Alberico D, Scott M E, Lautens M (2007). Chem Rev.

[11] Ackermann L, Vicente R, Kapdi A R (2009). Angew Chem, Int Ed.

[12] Daugulis O, Do H-Q, Shabashov D (2009). Acc Chem Res.

[13] Chen X, Engle K M, Wang D-H, Yu J-Q (2009). Angew Chem, Int Ed.

[14] Carrer A, Brion J-D, Messaoudi S, Alami M (2013). Org Lett.

[15] Li Q, Zhang S-Y, He G, Ai Z, Nack W A, Chen G (2014). Org Lett.

[16] Hull K L, Sanford M S (2009). J Am Chem Soc.

[17] Ackermann L, Lygin A V (2011). Org Lett.

[18] Zaitzev V G, Shabashov D, Daugulis O (2005). J Am Chem Soc.

[19] Dangel B D, Godula K, Youn S W, Sezen B, Sames D (2002). J Am Chem Soc.

[20] Hinman A, Du Bois J (2003). J Am Chem Soc.

[21] O’Malley S J, Tan K L, Watzke A, Bergman R G, Ellman J A (2005). J Am Chem Soc.

[22] Yamaguchi J, Yamaguchi A D, Itami K (2012). Angew Chem, Int Ed.

[23] Ramkumar N, Nagarajan R (2013). J Org Chem.

[24] Stuart D R, Villemure E, Fagnou K (2007). J Am Chem Soc.

[25] Liang Z, Zhao J, Zhang Y (2010). J Org Chem.

[26] Fan S, Chen Z, Zhang X (2012). Org Lett.

[27] Willis N J, Smith J M (2014). RSC Adv.

[28] Truong T, Alvarado J, Tran L D, Daugulis O (2010). Org Lett.

[29] Mao Z, Wang Z, Xu Z, Huang F, Yu Z, Wang R (2012). Org Lett.

[30] Xi P, Yang F, Qin S, Zhao D, Lan J, Gao G, Hu C, You J (2010). J Am Chem Soc.

[31] Nishino M, Hirano K, Satoh T, Miura M (2012). Angew Chem, Int Ed.

[32] Grimster N P, Gauntlett C, Godfrey C R A, Gaunt M J (2005). Angew Chem, Int Ed.

[33] Jiang H, Feng Z, Wang A, Liu X, Chen Z (2010). Eur J Org Chem.

[34] Ackermann L, Jeyachandran R, Potukuchi H K, Novak P, Büttner L (2010). Org Lett.

[35] Meng G, Niu H-Y, Qu G-R, Fossey J S, Li J-P, Guo H-M (2012). Chem Commun.

[36] Dwight T A, Rue N R, Charyk D, Josselyn R, DeBoef B (2007). Org Lett.

[37] Pintori D G, Greaney M F (2011). J Am Chem Soc.

[38] Reddy V P, Iwasaki T, Kambe N (2013). Org Biomol Chem.

[39] Agalave S G, Maujan S R, Pore V S (2011). Chem – Asian J.

[40] Shafran E A, Bakulev V A, Rozin Yu A, Shafran Yu M (2008). Chem Heterocycl Compd.

[41] Panteleev J, Geyer K, Aguilar-Aguilar A, Wang L, Lautens M (2010). Org Lett.

[42] Jiao L-Y, Oestreich M (2013). Chem – Eur J.

[43] Wu Y, Wang J, Mao F, Kwong F Y (2014). Chem – Asian J.

[44] Lafrance M, Fagnou K (2006). J Am Chem Soc.

[45] Ackermann L, Vicente R, Althammer A (2008). Org Lett.

[46] Ackermann L, Novák P (2009). Org Lett.

[47] Lapointe D, Fagnou K (2010). Chem Lett.

[48] Rousseaux S, Gorelsky S I, Chung B K, Fagnou K (2010). J Am Chem Soc.

[49] Blake A J, Clark B A J, McNab H, Sommerville C C (1997). J Chem Soc, Perkin Trans 1.

[50] Ponpandian T, Muthusubramanian S (2012). Tetrahedron Lett.

