# Supplementary material for: Sequential decarboxylative azide–alkyne cycloaddition and dehydrogenative coupling reactions: one-pot synthesis of polycyclic fused triazoles
Source: Beilstein J Org Chem. 2014 Dec 17;10:3031–7. doi: 10.3762/bjoc.10.321 (PMC4311670; doi:10.3762/bjoc.10.321)
Supplement: File 1 — X-ray crystallographic data of 4f, characterization, 1H and 13C NMR data of compounds 3a and 4a–m. [file Beilstein_J_Org_Chem-10-3031-s001.pdf]

Supporting Information

for

Sequential decarboxylative azide–alkyne cycloaddition and dehydrogenative coupling reactions: one-pot synthesis of polycyclic fused triazoles

Kuppusamy Bharathimohan[1,2], Thanasekaran Ponpandian[3], A. Jafar Ahamed[*1] and Nattamai Bhuvanesh[4]

Address: [1]PG and Research Department of Chemistry, Jamal Mohamed College, affiliated to the Bharathidasan university, Thiruchirapalli - 620020, Tamilnadu, India, [2]Orchid Chemicals & Pharmaceuticals Ltd, Drug Discovery Research, R&D Center, Sholinganallur, Chennai - 600119, India [3]Inogent Laboratories Pvt Ltd, API R&D, 28A, IDA, Nacharam, Hyderabad-500076, India and [4]X-ray Diffraction Laboratory, Department of Chemistry, Texas A&M University, College Station, Texas 77842, United States.

Email: A. Jafar Ahamed* - agjafar@yahoo.co.in
*Corresponding author

X-ray crystallographic data of 4f, characterization, ^{1}H and ^{13}C NMR data of compounds 3a and 4a–m

Contents

X-ray crystallographic data of **4f**…………………………………………………….....S2–S4

Experimental and Characterization data of **3a** and **4a**–**4m**……………………………… S4–S8

^{1}H and ^{13}C spectra of **3a** and **4a–4m**……………………………………………………......S9–S22

X-ray crystallographic data of 4f

Data Collection

A Leica MZ 7.5 microscope was used to identify a suitable colorless column with very well defined faces with dimensions (max, intermediate, and min) 0.35 mm x 0.12 mm x 0.10 mm from a representative sample of crystals of the same habit. The crystal mounted on a nylon loop was then placed in a cold nitrogen stream (Oxford) maintained at 110 K.

A BRUKER APEX 2 X-ray (three-circle) diffractometer was employed for crystal screening, unit cell determination, and data collection. The goniometer was controlled using the APEX2 software suite, v2008-6.0. The sample was optically centered with the aid of a video camera such that no translations were observed as the crystal was rotated through all positions. The detector was set at 6.0 cm from the crystal sample (APEX2, 512x512 pixel). The X-ray radiation employed was generated from a Mo sealed X-ray tube (K_{α} = 0.70173Å with a potential of 40 kV and a current of 40 mA) fitted with a graphite monochromator in the parallel mode (175 mm collimator with 0.5 mm pinholes).

60 data frames were taken at widths of 0.5°. These reflections were used in the auto-indexing procedure to *determine* the unit cell. A suitable cell was found and refined by nonlinear least squares and Bravais lattice procedures. The unit cell was verified by examination of the *h k l* overlays on several frames of. No super-cell or erroneous reflections were observed.

After careful examination of the unit cell, a standard data collection procedure was initiated using omega scans.

Data Reduction, Structure Solution, and Refinement

Integrated intensity information for each reflection was obtained by reduction of the data frames with the program APEX2. The integration method employed a three dimensional profiling algorithm and all data were corrected for Lorentz and polarization factors, as well as for crystal decay effects. Finally the

data was merged and scaled to produce a suitable data set. The absorption correction program SADABS was employed to correct the data for absorption effects and systematic errors.

Systematic reflection conditions and statistical tests of the data suggested the space group $P2_1/c$. A solution was obtained readily using SHELXTL (XS). Hydrogen atoms were placed in idealized positions and were set riding on the respective parent atoms. All non-hydrogen atoms were refined with anisotropic thermal parameters. Absence of additional symmetry or solvent accessible voids was confirmed using PLATON (ADDSYM). The structure was refined (weighted least squares refinement on F^2) to convergence.

Olex2 was employed for the final data presentation and structure plots.

Table 1. Crystal data and structure refinement for JAB_KB_131009_A2_1.

Identification code	jab	
Empirical formula	C16 H11 N5	
Formula weight	273.30	
Temperature	296.15 K	
Wavelength	0.71073 Å	
Crystal system	Monoclinic	
Space group	P 1 21/c 1	
Unit cell dimensions	a = 8.249(2) Å	α= 90°.
	b = 5.6691(14) Å	β= 96.319(3)°.
	c = 26.797(7) Å	γ = 90°.
Volume	1245.5(5) Å^3	
Z	4	
Density (calculated)	1.457 Mg/m^3	
Absorption coefficient	0.093 mm^{-1}	
F(000)	568	
Crystal size	0.35 x 0.12 x 0.1 mm^3	
Theta range for data collection	1.529 to 27.477°.	
Index ranges	-10<=h<=10, -7<=k<=7, -34<=l<=34	
Reflections collected	13968	
Independent reflections	2837 [R(int) = 0.0509]	
Completeness to theta = 25.242°	99.9 %	

Absorption correction	Semi-empirical from equivalents
Max. and min. transmission	0.7458 and 0.6401
Refinement method	Full-matrix least-squares on F^2
Data / restraints / parameters	2837 / 0 / 191
Goodness-of-fit on F^2	1.058
Final R indices [I>2sigma(I)]	R1 = 0.0425, wR2 = 0.0967
R indices (all data)	R1 = 0.0600, wR2 = 0.1072
Extinction coefficient	n/a
Largest diff. peak and hole	0.242 and -0.246 e.$Å^{-3}$

Experimental and Characterization data of 3a and 4a–4m

General information:

All the reagents and solvents were purchased from the commercial sources. All ^{1}H NMR and ^{13}C NMR spectra were recorded at 400 and 100 MHz respectively, with TMS as internal standard Chemical shifts are reported in parts per million (δ) relative to TMS, coupling constants (J values) were reported in Hertz (Hz). Infrared spectra were recorded on a Shimadzu FT-IR instrument (KBr pellet) and the band positions are reported in reciprocal of centimeters (cm^{-1}). Melting points were determined on a melting point apparatus (Inlab Pvt Ltd, India) equipped with a thermometer and were uncorrected. Elemental analyses were performed on a Perkin-Elmer 2400 Series II Elemental CHNS analyzer. Column chromatography was performed using silica gel (60-120 mesh). It is to be noted that in the C-13 spectrum of **4**, not all the carbons are picking up because of its poor solubility.

General procedure for the synthesis of fused triazolo-quinoxaline derivatives (**4**).

Substituted phenylpropiolic acids (**2**) were prepared by the literature procedure. To a mixture of 1-(2-azidophenyl)-1*H*-benzo[*d*]imidazole **(1a)** or 1-(2-azidophenyl)-1*H*-imidazole (**1b**) (0.85 mmol), 2-alkynoic acid **(2)** (1.02 mmol) and $Cu(OAc)_2{\cdot}H_2O$ (0.085 mmol, 10 mol%) in toluene (8 mL) was added to sodium ascorbate (0.17 mmol, 20 mol%) at room temperature. The mixture was stirred at 80 ^oC for 2h. $Cu(OAc)_2{\cdot}H_2O$ (1.7 mmol), $Pd(OAc)_2$ (0.043 mmol, 5 mol%) and pivalic acid (2.55 mmol) were added into above reaction mixture and then refluxed at 120 ^oC for 3 h. The reaction mixture was cooled to room temperature and diluted with ethyl acetate (200 mL). The mixture was filtered through a celite pad and the filtrate was washed with water, dried over anhydrous Na_2SO_4 and concentrated under vacuum. The residue was purified by column chromatography using hexane/ethyl acetate as eluent to obtain the desired product **4** (60-97%).

1-(2-(4-Phenyl-1*H*-1,2,3-triazol-1-yl)phenyl)-1*H*-benzo[*d*]imidazole (3a): Light yellow color solid; mp 169-171 ^oC; IR (KBr) 3428, 3052, 2924, 1620, 1478 cm^{-1}; ^1H NMR (400 MHz, $CDCl_3$) δ 6.99-7.23 (m, 3H), 7.27-7.33 (m, 4H), 7.5 (d, *J* = 6.9 Hz, 2H), 7.66 (t, *J* =4.4 Hz, 1H), 7.72-7.77 (m, 2H), 7.83 (d, *J* =6.6 Hz, 2H), 7.97-8.0 (m, 1H); ^{13}C NMR (100 MHz, $CDCl_3$): δ 109.7, 119.6, 120.9, 123.3, 124.5, 125.9, 127.3, 128.4, 128.5, 128.7, 129.5, 130.3, 130.8, 133.4, 148.5; Anal. Calcd. for $C_{21}H_{15}N_5$: C, 74.76; H, 4.48; N, 20.76. found C, 74.71; H, 4.39; N, 20.85; ESI-MS (M + 1) 338.1.

1-Phenylbenzo[4,5]imidazo[1,2-*a*][1,2,3]triazolo[5,1-*c*]quinoxaline (4a): White solid; mp 248-251 ^oC; IR (KBr) 3435, 3058, 2923, 1625, 1488 cm^{-1}; ^1H NMR (400 MHz, $CDCl_3$) δ 7.48-7.56 (m, 3H), 7.60 (t, *J* = 7.6 Hz, 3H), 7.71 (t, *J* = 7.7 Hz, 1H), 8.04-8.06 (m, 1H), 8.23-8.26 (m, 1H), 8.49 (d, *J* = 8.36 Hz, 1H), 8.8 (d, *J* = 8.08 Hz, 1H), 8.85 (d, *J* = 7.56 Hz, 2H); ^{13}C NMR (100 MHz, $CDCl_3$): δ 113.1, 115.9, 118.0, 120.4, 121.7, 123.6, 124.8, 125.0, 126.1, 126.3, 128.6, 128.7, 129.0, 129.2, 129.7, 130.9, 139.5, 144.3, 145.1; Anal. Calcd. for $C_{21}H_{13}N_5$: C, 75.21; H, 3.91; N, 20.88. found C, 75.27; H, 3.94; N, 20.84; ESI-MS (M + 1) 336.1.

1-(4-Methoxyphenyl)benzo[4,5]imidazo[1,2-*a*][1,2,3]triazolo[5,1-*c*]quinoxaline (4b): Light yellow color solid; mp 205-208 ^{o}C; IR (KBr) 3429, 3062, 2948, 1629, 1477 cm^{-1}; ^{1}H NMR (400 MHz, $CDCl_3$) δ 3.94 (s, 3H), 7.14 (d, J = 8.8 Hz, 2H),7.54-7.57 (m, 2H), 7.6 (t, J = 7.8 Hz, 1H), 7.72 (t, J = 7.72 Hz, 1H), 8.06-8.08 (m, 1H), 8.26-8.28 (m, 1H), 8.51 (d, J = 8.44 Hz, 1H), 8.8-8.81 (m, 3H); ^{13}C NMR (100 MHz, DMSO-d_6): δ 55.2, 113.8, 113.9, 116.7, 116.8, 120.4, 122, 124.5, 124.6, 125.9, 126.1, 129.3, 129.5, 143.3, 143.5, 159.8; Anal. Calcd. for $C_{22}H_{15}N_5O$: C, 72.32; H, 4.14; N, 19.17. found C, 72.04; H, 4.19; N, 19.03; ESI-MS (M + 1) 366.1.

Methyl3-(benzo[4,5]imidazo[1,2-*a*][1,2,3]triazolo[5,1-*c*]quinoxalin-1-yl)benzoate (4c): White solid; mp 256-260 ^{o}C; IR (KBr) 3427, 3065, 2924, 1716 1540, 1445, 1283 cm^{-1}; ^{1}H NMR (400 MHz, $CDCl_3$) δ 4.03 (s, 3H), 7.57 (t, J = 3.92 Hz, 2H),7.63-7.72 (m, 2H), 7.76 (t, J = 7.88 Hz, 1H), 8.08 (d, J = 8.56 Hz, 1H), 8.17 (d, J = 7.64 Hz, 1H), 8.30 (d, J = 8.44 Hz, 1H), 8.55 (d, J = 8.4 Hz, 1H), 8.84 (d, J = 8.12 Hz, 1H), 9.13 (d, J = 7.68, 1H), 9.67 (s, 1H); ^{13}C NMR (100 MHz, CD_2Cl_2) δ 52.5, 113.7, 116.5, 118.2, 121.8, 123.9, 125.3, 125.4, 126.5, 126.8, 129.0, 129.6, 129.9, 130.2, 130.7, 131.1, 133.2, 167.3. Anal. Calcd. for $C_{23}H_{15}N_5O_2$: C, 70.22; H, 3.84; N, 17.80. found C, 70.14; H, 3.72; N, 17.72; ESI-MS (M + 1) 394.1

1-(Thiophen-2-yl)benzo[4,5]imidazo[1,2-*a*][1,2,3]triazolo[5,1-*c*]quinoxaline (4d): Pale brown color solid; mp 240-243 ^{o}C; IR (KBr) 3425, 3072, 2948, 1628, 1494, 1227 cm^{-1}; ^{1}H NMR (400 MHz, $CDCl_3$) δ 7.3 (t, J = 4 Hz, 1H), 7.49-7.64 (m, 4H), 7.73 (t, J = 7.84 Hz, 1H), 8.09-8.11 (m, 1H), 8.27-8.29 (m, 1H), 8.51 (d, J = 8.52 Hz, 1H), 8.79 (d, 8.2 Hz, 1H), 9.15 (d, J = 3.5 Hz, 1H); ^{13}C NMR (100 MHz, $CDCl_3$ & CD_3OD mixture) δ 112.1, 115.1, 116.9, 120.6, 123.9, 124.1, 125.2, 125.4, 126.1, 127.2, 128.2, 128.6, 129.8; Anal. Calcd. for $C_{19}H_{11}N_5S$: C, 66.81; H, 3.25; N, 20.51; S, 9.39. found C, 66.81; H, 3.28; N, 20.31; S, 9.33 ESI-MS (M + 1) 342.1.

1-Pentylbenzo[4,5]imidazo[1,2-*a*][1,2,3]triazolo[5,1-*c*]quinoxaline (4e): White solid; mp 146-149 ^{o}C; IR (KBr) 3431, 3123, 2926, 2858, 1536, 1501, 1418, 1217, 1103 cm^{-1}; ^{1}H NMR (400 MHz, $CDCl_3$) δ 0.91 (t, J = 7 Hz, 3H), 1.39-1.54 (m, 4H), 1.98-2.05 (m, 2H), 3.39 (t, J = 7.68 Hz, 2H) 7.52-7.61 (m, 3H), 7.7 (t, J = 7.8 Hz, 1H) 8.04-8.06 (m, 1H), 8.23-8.26 (m, 1H), 8.47 (d, J = 8.36 Hz, 1H), 8.74 (d, J = 8.08 Hz, 1H); ^{13}C NMR (100 MHz, DMSO-d_6) δ 13.8, 21.8, 25.0, 28.0, 30.7, 116.7, 116.9, 120.4, 121.2, 123.0, 124.4, 124.5, 125.9, 126.0, 129.2, 130.3, 139.5, 144.0, 144.9. Anal. Calcd. for $C_{20}H_{19}N_5$: C, 72.93; H, 5.81; N, 21.26. found C, 72.79; H, 5.88; N, 20.98; ESI-MS (M + 1) 330.1

1-Methylbenzo[4,5]imidazo[1,2-*a*][1,2,3]triazolo[5,1-*c*]quinoxaline (4f): White solid; mp 248-252 °C; IR (KBr) 3432, 3069, 2978, 1629, 1477 cm^{-1}; ^1H NMR (400 MHz, $CDCl_3$) δ 2.99 (s, 3H), 7.53-7.61 (m, 3H), 7.7 (t, *J* = 7.8 Hz, 1H) 8.03-8.05 (m, 1H), 8.22-8.25 (m, 1H), 8.46 (d, *J* = 8.4 Hz, 1H), 8.72 (d, *J* = 8.12 Hz, 1H); ^{13}C NMR (100 MHz, DMSO-d_6) δ 11.1, 113.8, 116.7, 116.9, 120.5, 124.3, 124.4, 126, 129.2; Anal. Calcd. for $C_{16}H_{11}N_5$: C, 70.32; H, 4.06; N, 25.63. found C, 70.18; H, 4.12; N, 25.39; ESI-MS (M + 1) 274.1

3-Phenylimidazo[1,2-*a*][1,2,3]triazolo[5,1-*c*]quinoxaline (4g): White solid; mp 255-259 °C; IR (KBr) 3432, 3059, 2927, 1623, 1479 cm^{-1}; ^1H NMR (400 MHz, $CDCl_3$) δ 7.43 (t, *J* = 7.4 Hz, 1H) 7.56-7.62 (m, 2H), 7.64-7.67 (m, 3H), 7.85-7.88 (m, 1H), 7.97-7.98 (d, *J* = 1.24 Hz, 1H); 8.75-8.8 (m, 3H); ^{13}C NMR (100 MHz, CD_2Cl_2, $CDCl_3$ & CD_3OD mixture) δ 114.1, 116.7, 118, 127.6, 128.5, 128.9, 129.2, 129.3, 132.6; Anal. Calcd. for $C_{17}H_{11}N_5$: C, 71.57; H, 3.89; N, 24.55. found C, 71.48; H, 3.83; N, 24.51; ESI-MS (M + 1) 286.1

Methyl 3-(imidazo[1,2-*a*][1,2,3]triazolo[5,1-*c*]quinoxalin-3-yl)benzoate (4h): Light white solid; mp 250-252 °C; IR (KBr) 3428, 3065, 2924, 1718 1540, 1439, 1282 cm^{-1}; ^1H NMR (400 MHz, $CDCl_3$) δ 3.99 (s, 3H) 7.65-7.72 (m, 4H) 7.89-7.91 (m, 1H) 8.00 (s, 1H) 8.12-8.14 (d, *J* = 7.72 Hz, 1H) 8.78-8.8 (m, 1H) 9.09 (d, *J* = 7.84 Hz, 1H) 9.49 (s, 1H); ^{13}C NMR (100 MHz, CD_2Cl_2 & CD_3OD mixture) δ 50.1, 114.4, 116.8, 118.1, 127.7, 129.1, 129.4, 129.5, 129.9, 131, 132.7, 132.9; Anal. Calcd. for $C_{19}H_{13}N_5O_2$: C, 66.47; H, 3.82; N, 20.40; O, 9.32. found C, 66.39; H, 3.75; N, 20.14; ESI-MS (M + 1) 344.1

3-(4-Methoxyphenyl)imidazo[1,2-*a*][1,2,3]triazolo[5,1-*c*]quinoxaline (4i): Light yellow color solid; mp 208-212 °C; IR (KBr) 3427, 3068, 2943, 1627, 1468 cm^{-1}; ^1H NMR (400 MHz, $CDCl_3$) δ 3.90 (s, 3H) 7.1-7.12 (d, *J* = 8.8 Hz, 2H) 7.61-7.68 (m, 3H) 7.84-7.87 (m, 1H) 7.95-7.96 (m, 1H) 8.72-8.76 (m, 3H); ^{13}C NMR (100 MHz, CD_2Cl_2) δ 55.7, 114.2, 116.4, 116.5, 118, 123.1, 124.3, 124.4, 127.4, 127.5, 129, 129.1, 129.9, 132.5,132.9, 135.6, 142.6, 160.5; Anal. Calcd. for $C_{18}H_{13}N_5O$: C, 68.56; H, 4.16; N, 22.21; O, 5.07 found C, 68.41; H, 4.18; N, 22.03; ESI-MS (M + 1) 316.1.

3-(Thiophen-2-yl)imidazo[1,2-*a*][1,2,3]triazolo[5,1-*c*]quinoxaline (4j): Light pink color solid; mp 238-240 °C; IR (KBr) 3429, 3069, 2946, 1627, 1489, 1224 cm^{-1}; ^1H NMR (400 MHz, $CDCl_3$) δ 7.43 (d, *J* = 4.84 Hz, 1H) 7.61-7.71 (m, 4H), 7.86 (d, *J* = 7.76 Hz, 1H), 7.97 (s, 1H),

8.73 (d, J = 7.88 Hz, 1H); 8.89 (d, J =2.92, 1H); ^{13}C NMR (100 MHz, CD_2Cl_2 and CD_3OD mixture) δ 114.1, 116.8, 118, 124.1, 124.6, 126.6, 127.6, 128.3, 128.9, 129.4, 132.6, 132.8; Anal. Calcd. for $C_{15}H_9N_5S$: C, 61.84; H, 3.11; N, 24.04; S, 11.01 found C, 61.79; H, 3.19; N, 24.1; S, 10.97; ESI-MS (M + 1) 392.1

3-Pentylimidazo[1,2-*a*][1,2,3]triazolo[5,1-*c*]quinoxaline (4k): White solid; mp 102-104 ^{o}C; IR (KBr) 3431, 3123, 2926, 2856, 1536, 1501, 1329, 1217, 1104 cm^{-1}; ^{1}H NMR (400 MHz, $CDCl_3$) δ 0.88 (t, J = 6.96 Hz, 3H), 1.37-1.49 (m, 4H), 1.92-2.0 (m, 2H), 3.28 (t, J = 7.68 Hz, 2H) 7.58-7.65 (m, 3H), 7.84 (d, J = 7.64 Hz, 1H) 7.9 (s, 1H) 8.69 (d, J = 7.64 Hz, 1H); ^{13}C NMR (100 MHz, CD_2Cl_2) δ 14.2, 22.9, 26, 29.1, 31.8, 113.1, 116.6, 117.7, 122.2, 124.3, 124.5, 127.2, 128.7, 132.9, 135.7, 143.8; Anal. Calcd. for $C_{16}H_{17}N_5$: C, 68.79; H, 6.13; N, 25.07. found C, 68.65; H, 6.19; N, 25.12; ESI-MS (M + 1) 280.1

3-Methylimidazo[1,2-*a*][1,2,3]triazolo[5,1-*c*]quinoxaline (4l): White solid; mp 210-214 ^{o}C; IR (KBr) 3429, 3071, 2974, 1626, 1472 cm^{-1}; ^{1}H NMR (400 MHz, $CDCl_3$) δ 2.9 (s, 3H) 7.58-7.66 (m, 3H), 7.84 (d, J = 7.88 Hz, 1H) 7.9 (s, 1H) 8.68 (d, J = 7.76 Hz, 1H); ^{13}C NMR (100 MHz, $CDCl_3$) δ 11.4, 112.8, 116.2, 117.7, 124.0, 124.1, 127.1, 128.6, 132.8. Anal. Calcd. for $C_{12}H_9N_5$: C, 64.56; H, 4.06; N, 31.37 found C, 64.43; H, 3.98; N, 30.92; ESI-MS (M + 1) 224.1.

3-Pentyl-5,6-diphenylimidazo[1,2-*a*][1,2,3]triazolo[5,1-*c*]quinoxaline (4m): White solid; mp 187-189 ^{o}C; IR (KBr) 3438, 3125, 2929, 2849, 1534, 1506, 1412, 1218, 1106 cm^{-1}; ^{1}H NMR (400 MHz, $CDCl_3$) δ 0.93 (t, J = 7 Hz, 3H), 1.46-1.61 (m, 4H), 2.02-2.06 (m, 2H), 3.38 (t, J = 7.64 Hz, 2H) 7.07 (d, J = 8.6 Hz, 1H), 7.15 (t, J = 7.64 Hz, 1H) 7.23 (d, J = 7.64 Hz, 2H), 7.43 (t, J = 7.76 Hz, 1H) 7.55-7.66 (m, 8H), 8.68 (d, J = 8.08 Hz, 1H); ^{13}C NMR (100 MHz, $CDCl_3$) δ 14.2, 22.6, 25.9, 28.7, 31.6, 117.5, 117.8, 121.6, 124.5, 125.5, 125.7, 126.4, 127.5, 127.7, 128.3, 130.1, 130.2, 131.6, 131.7, 133.6, 135.3, 142.0, 144.3. Anal. Calcd. for $C_{28}H_{25}N_5$: C, 77.93; H, 5.84; N, 16.23. found C, 77.95; H, 5.81; N, 15.99; ESI-MS (M + 1) 432.1

^{1}H and ^{13}C spectra of 3a and 4a–4m

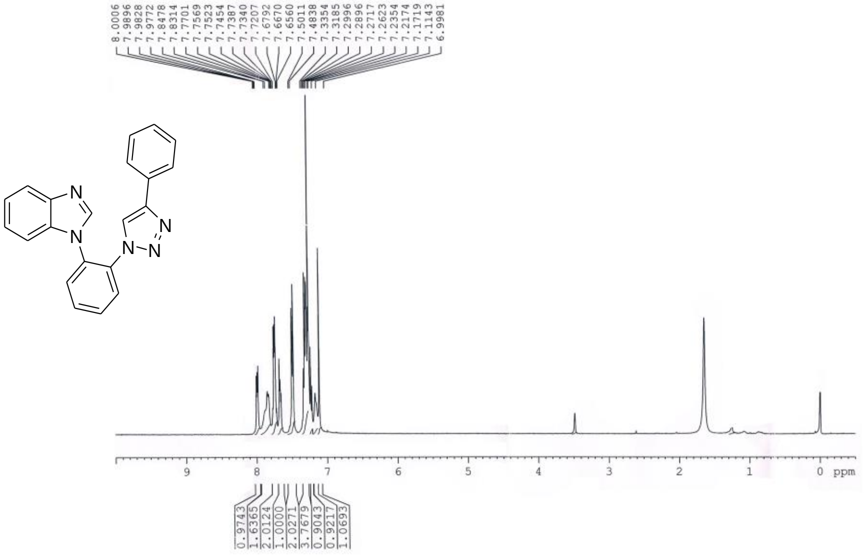

^{1}H NMR spectrum of **3a**

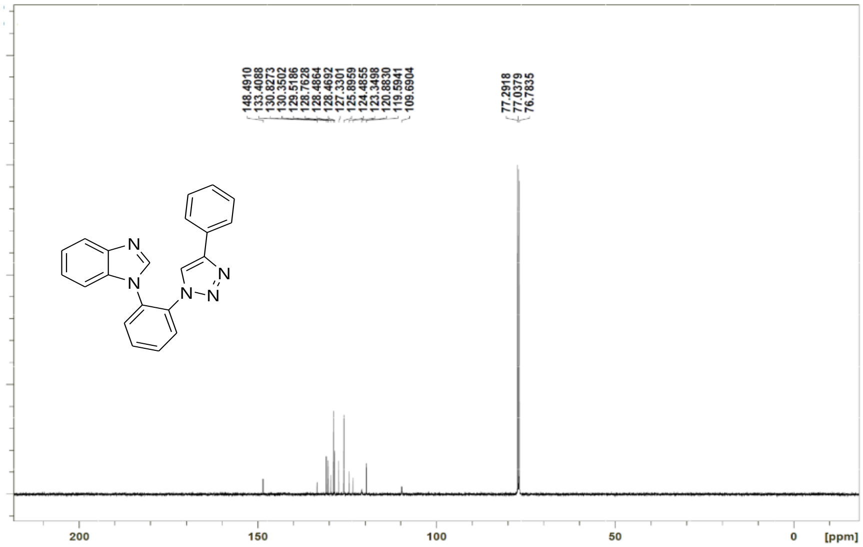

^{13}C NMR spectrum of **3a**

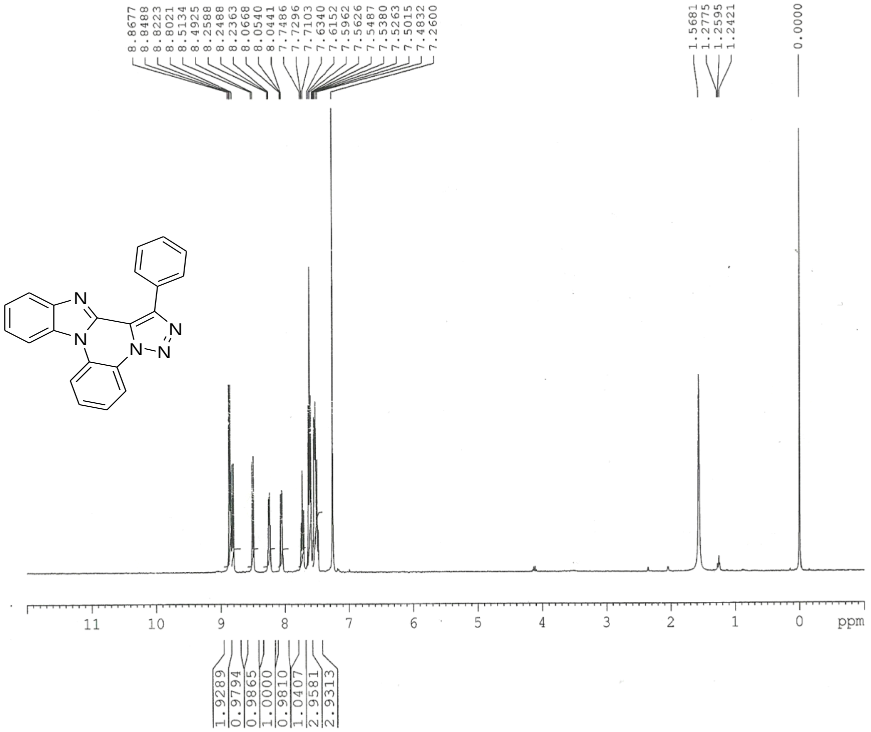

^{1}H NMR spectrum of **4a**

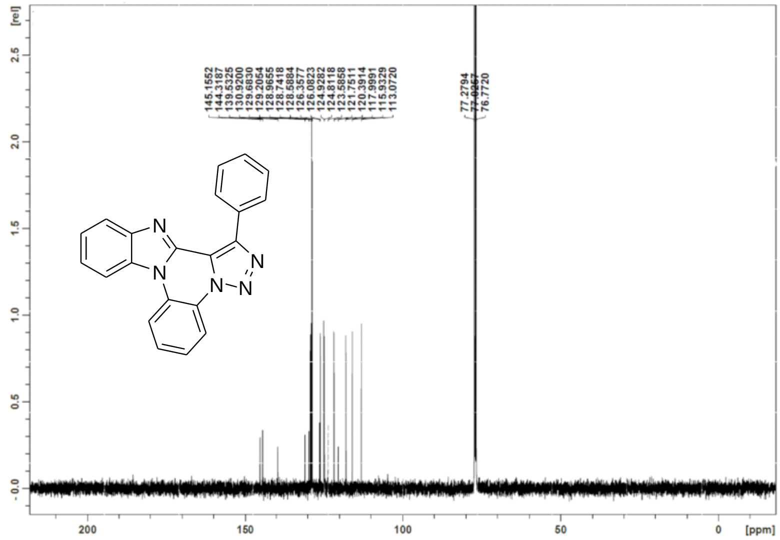

^{13}C NMR spectrum of **4a**

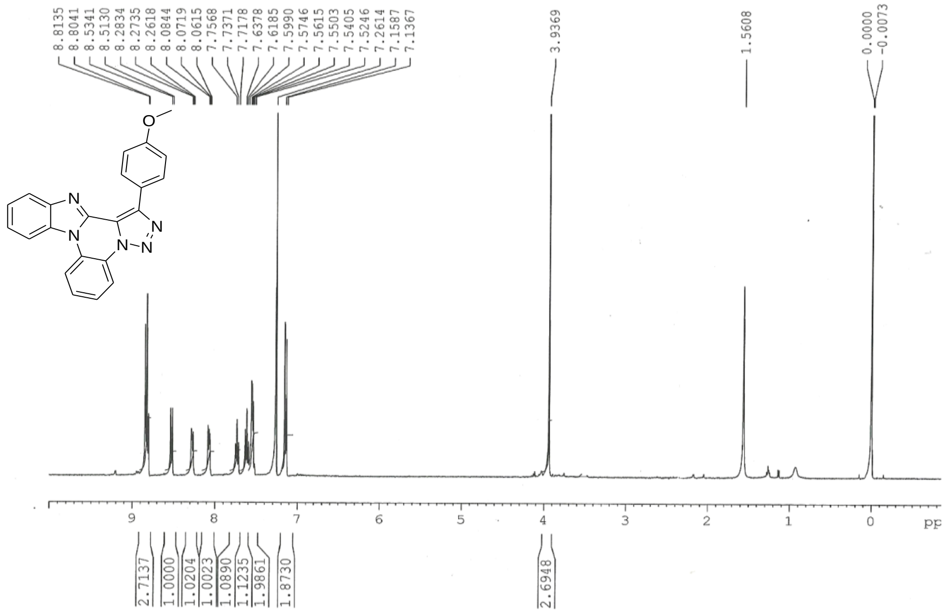

^{1}H NMR spectrum of **4b**

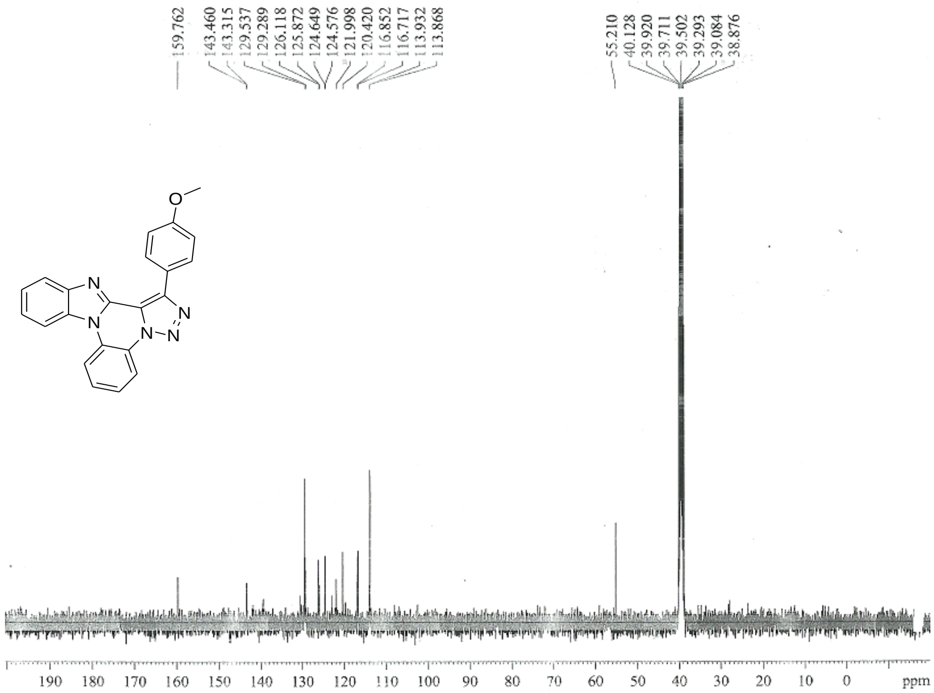

^{13}C NMR spectrum of **4b**

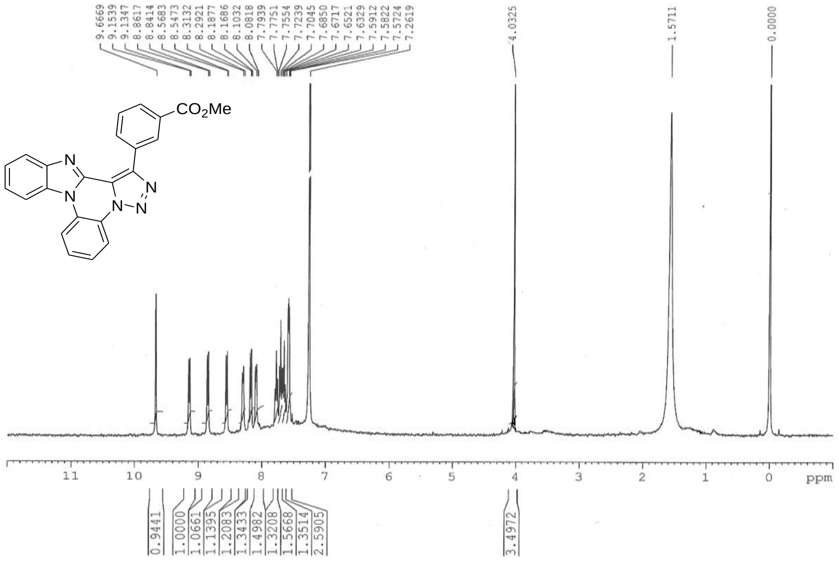

^{1}H NMR spectrum of **4c**

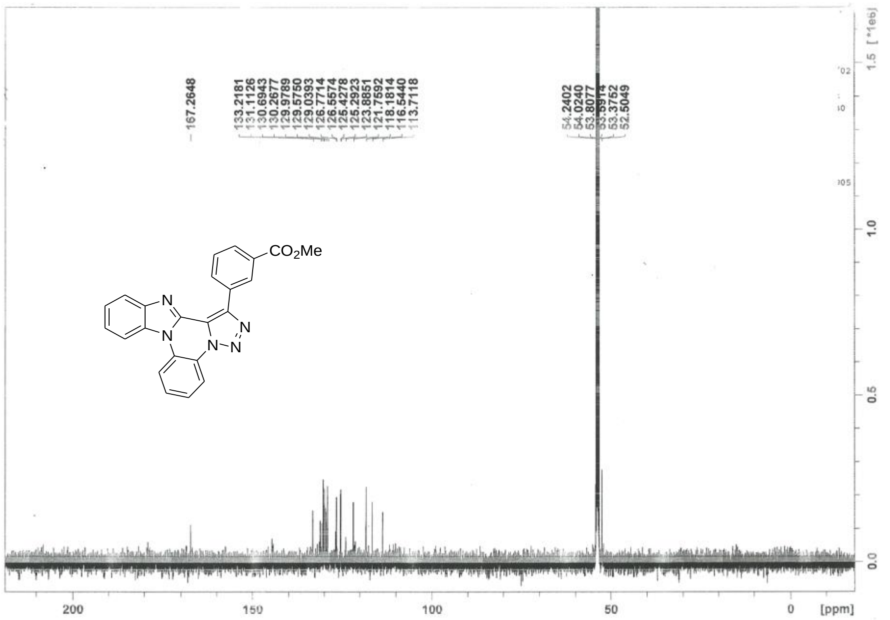

^{13}C NMR spectrum of **4c**

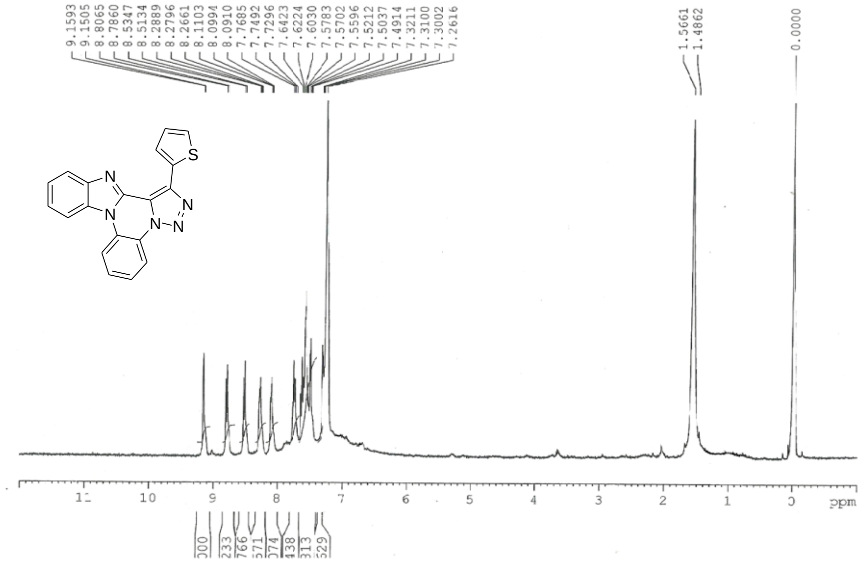

^{1}H NMR spectrum of **4d**

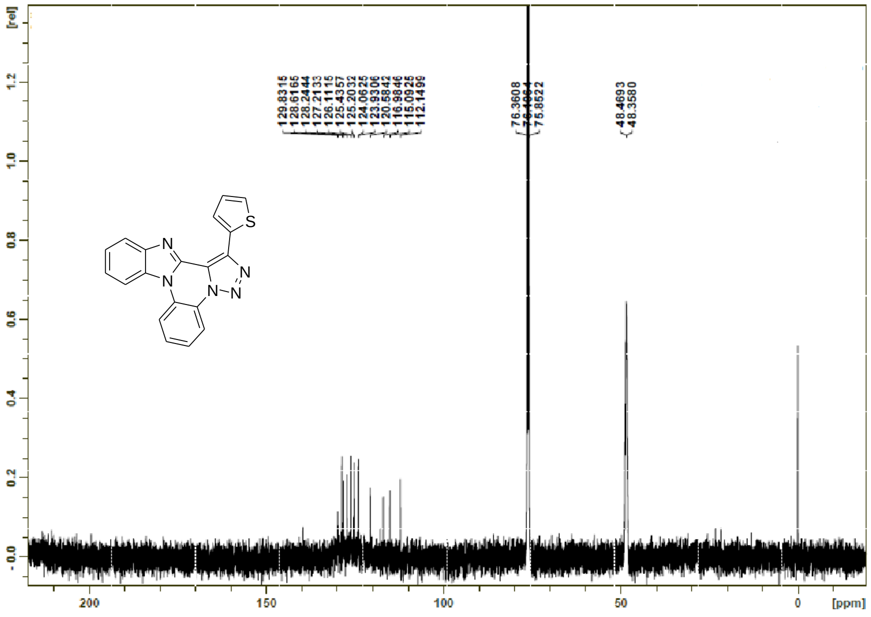

^{13}C NMR spectrum of **4d**

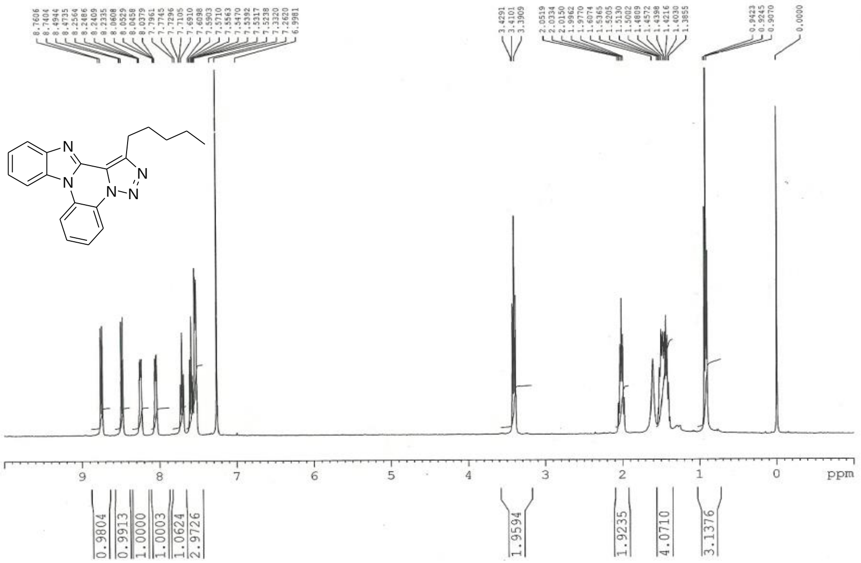

^{1}H NMR spectrum of **4e**

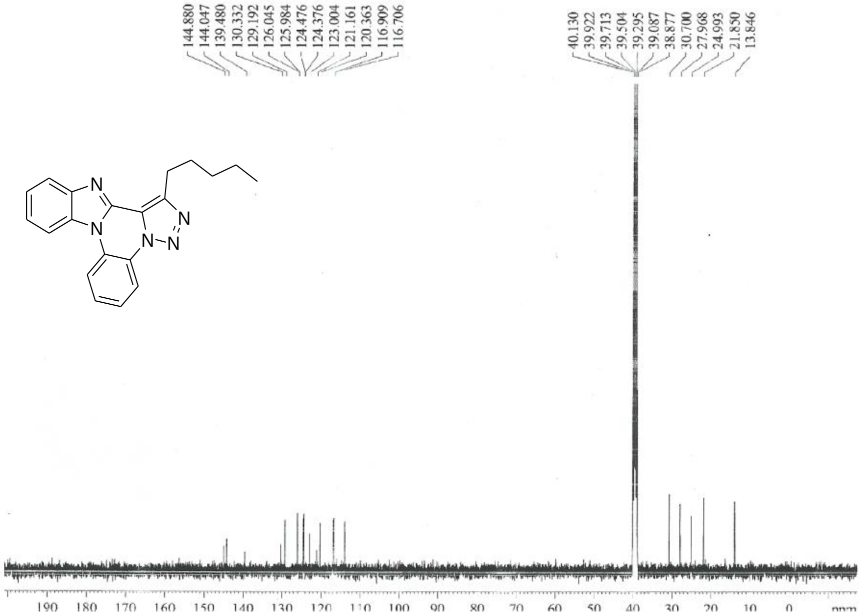

^{13}C NMR spectrum of **4e**

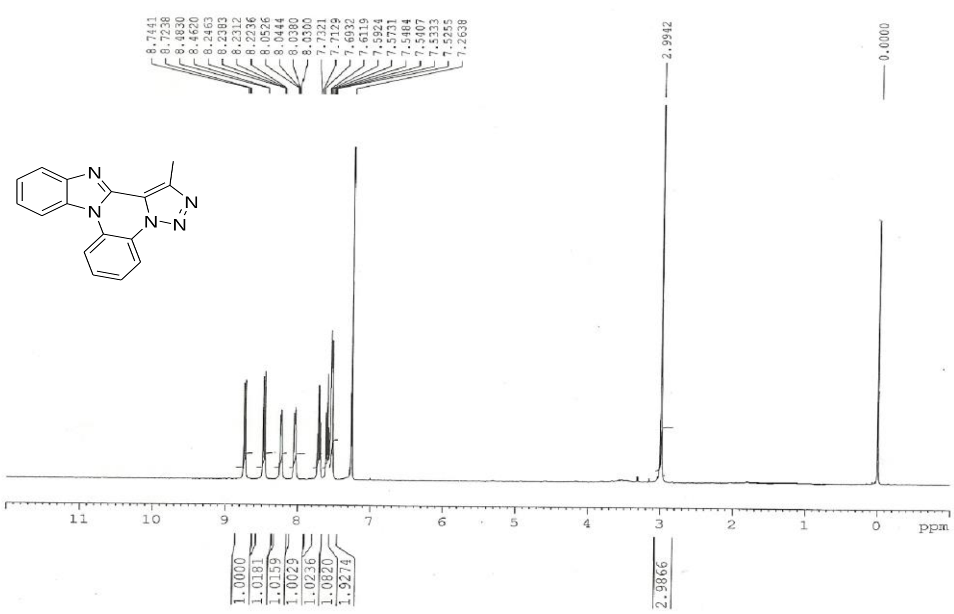

^{1}H NMR spectrum of **4f**

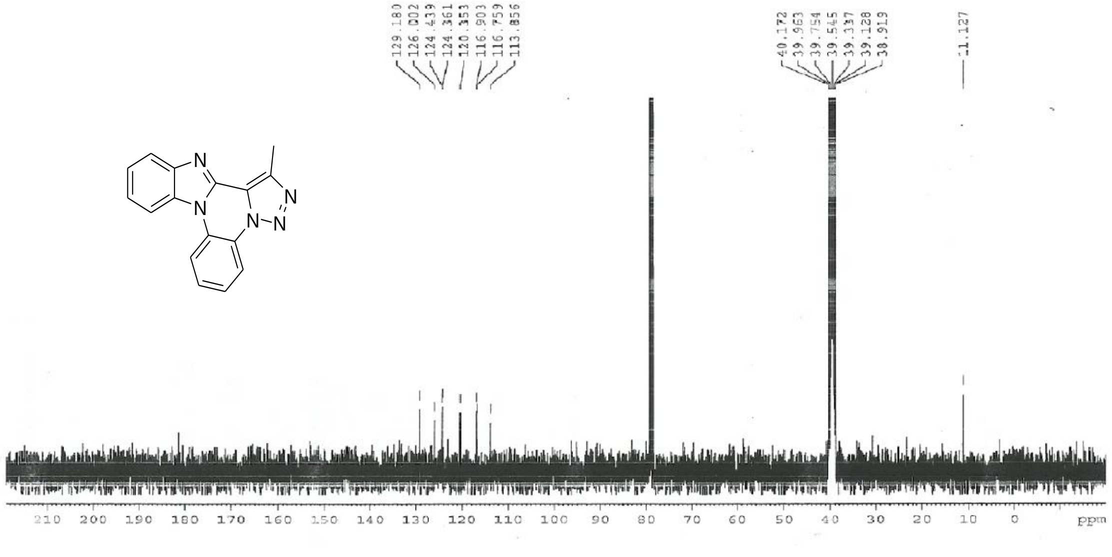

^{13}C NMR spectrum of **4f**

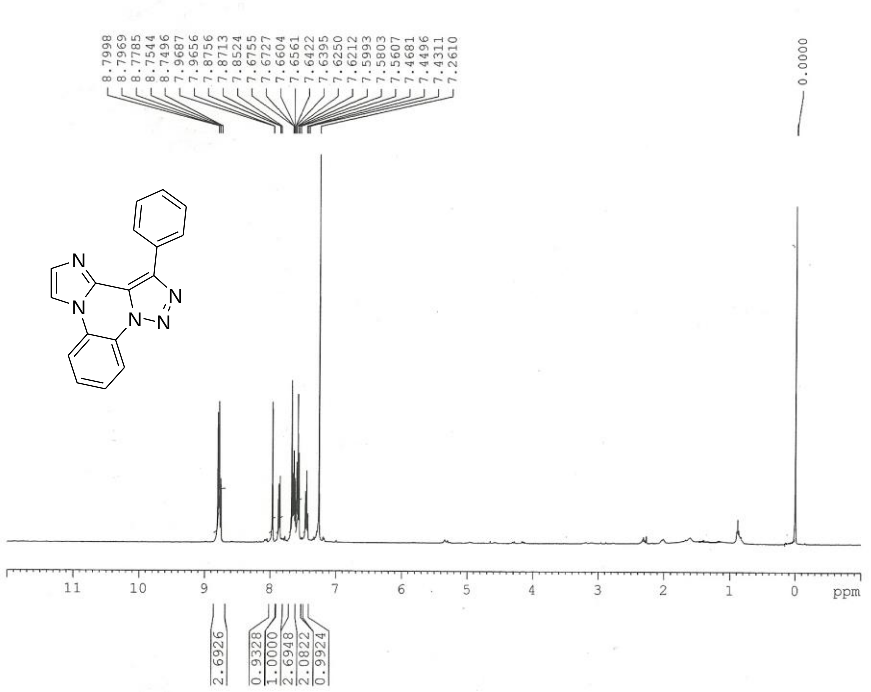

^{1}H NMR spectrum of **4g**

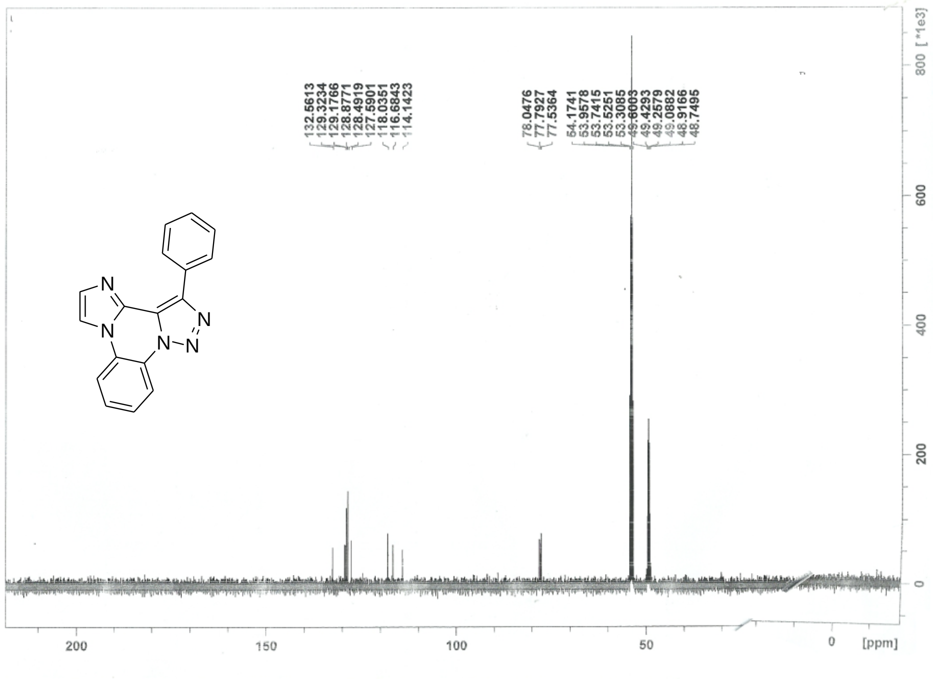

^{13}C NMR spectrum of **4g**

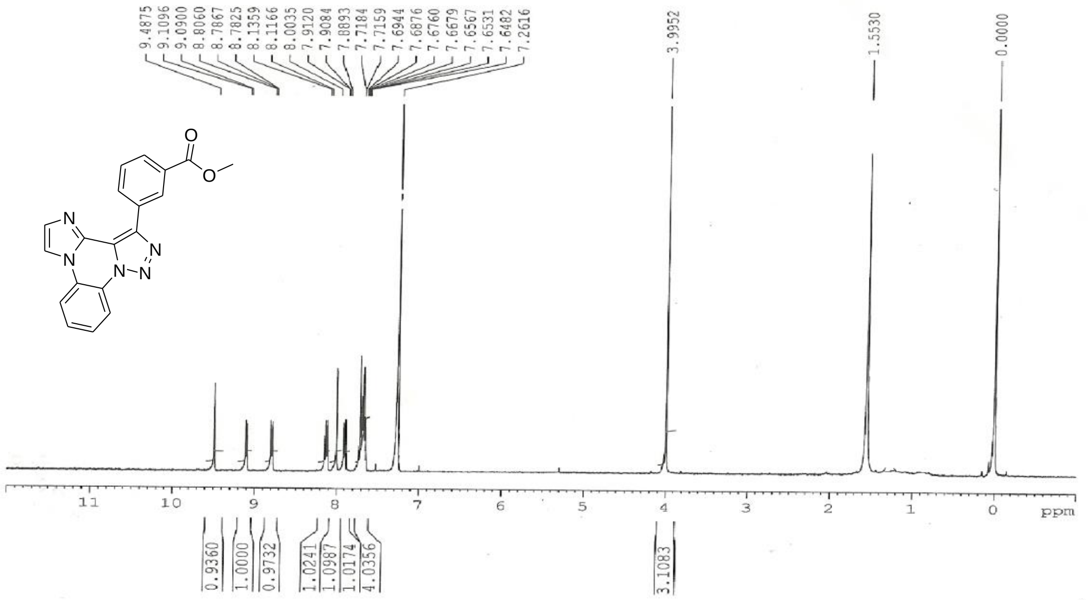

^{1}H NMR spectrum of **4h**

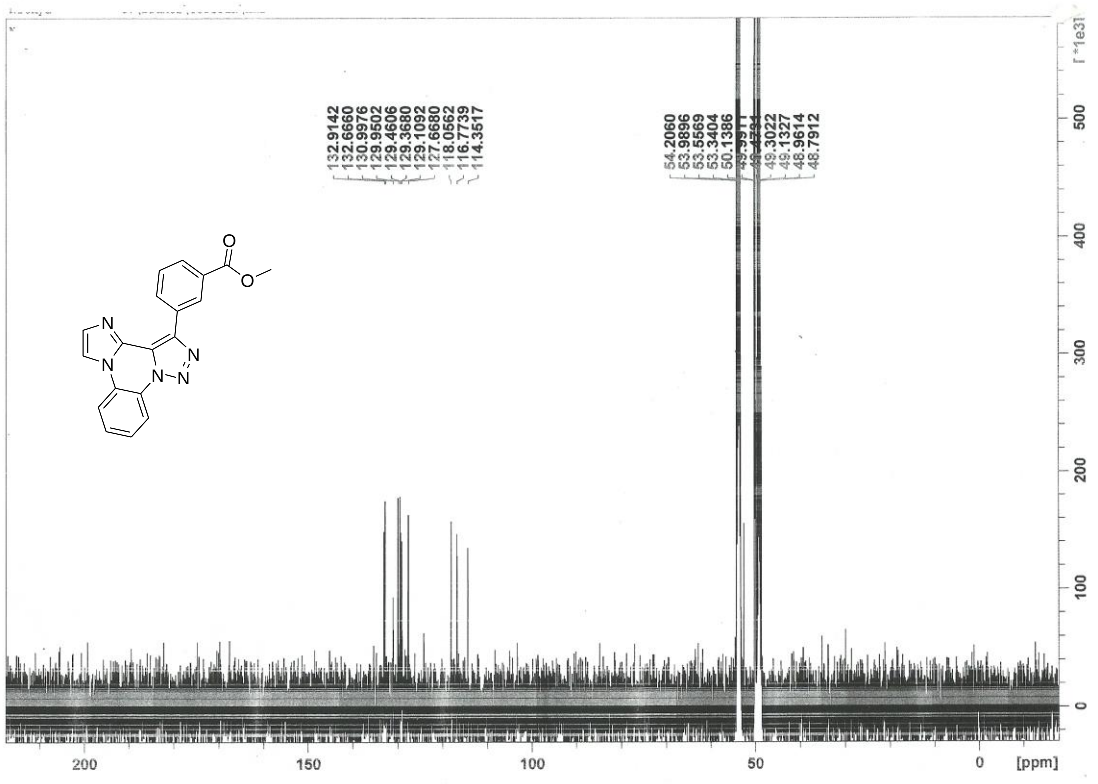

^{13}C NMR spectrum of **4h**

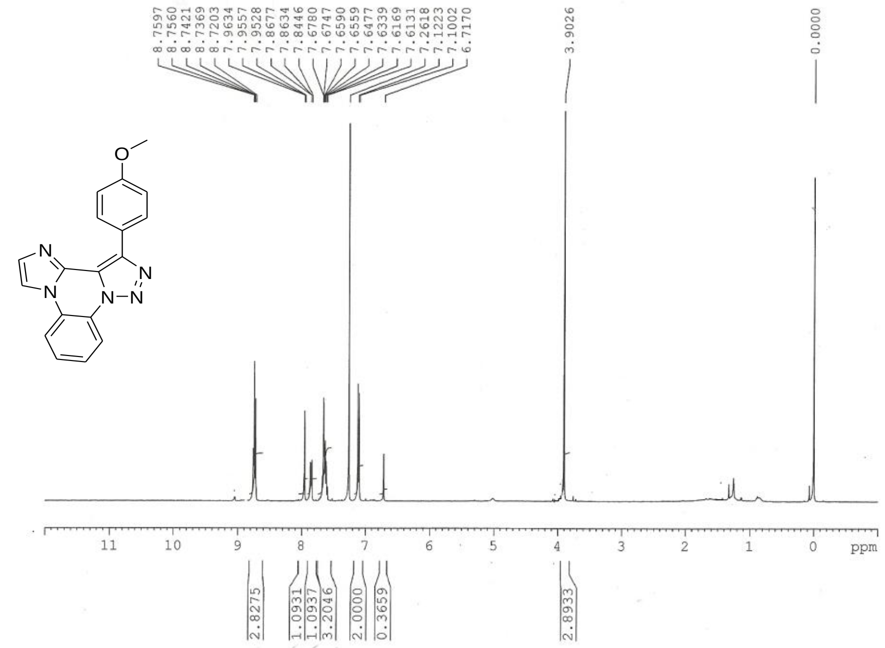

^{1}H NMR spectrum of **4i**

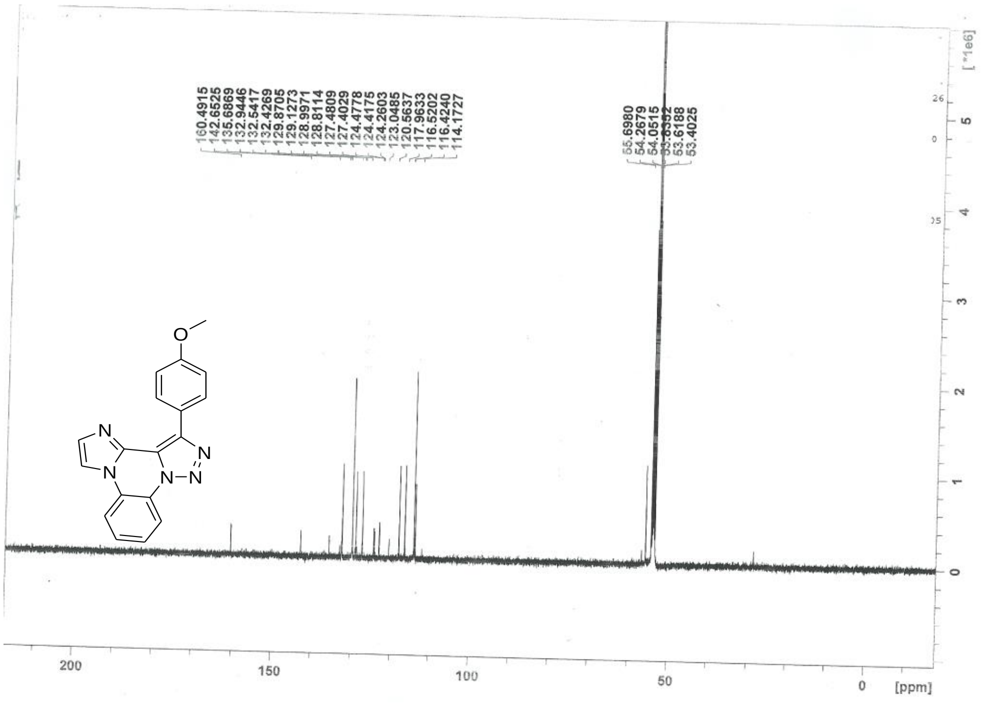

^{13}C NMR spectrum of **4i**

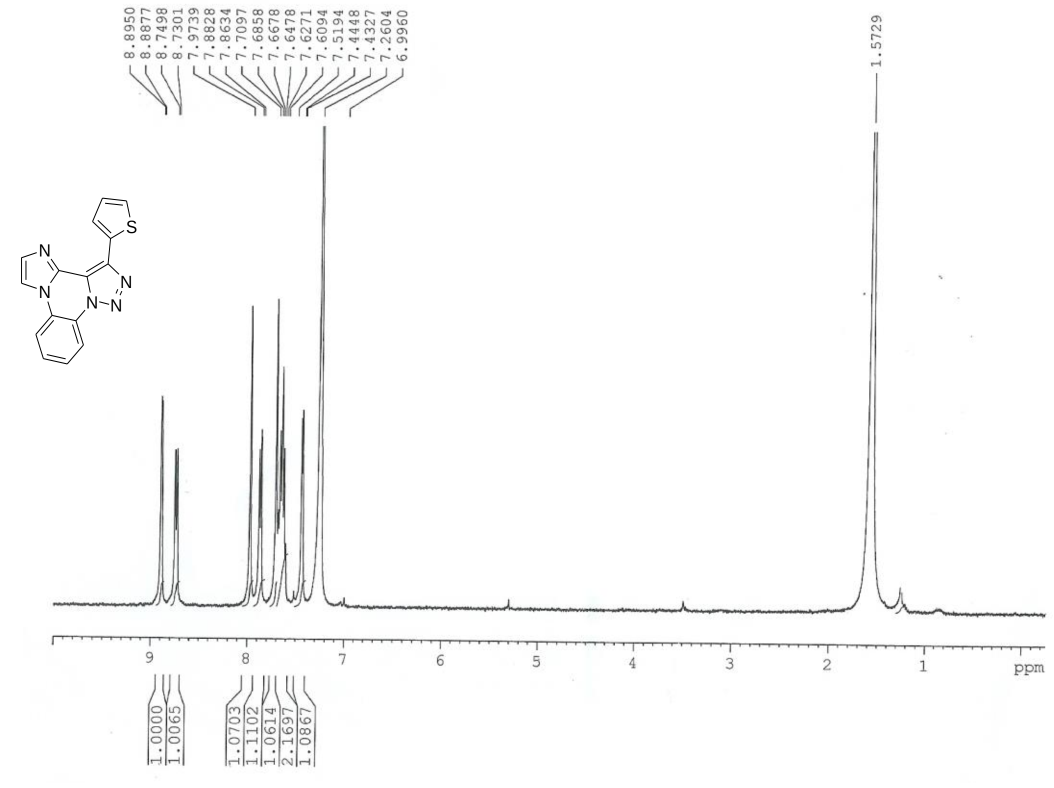

^{1}H spectrum of **4j**

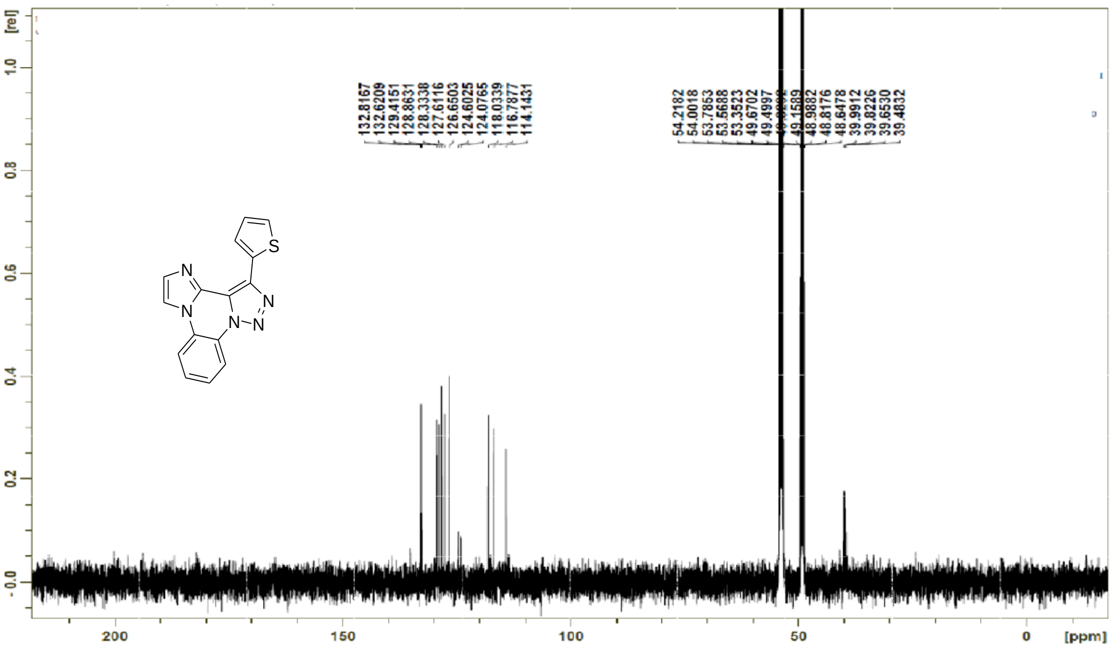

^{13}C NMR spectrum of **4j**

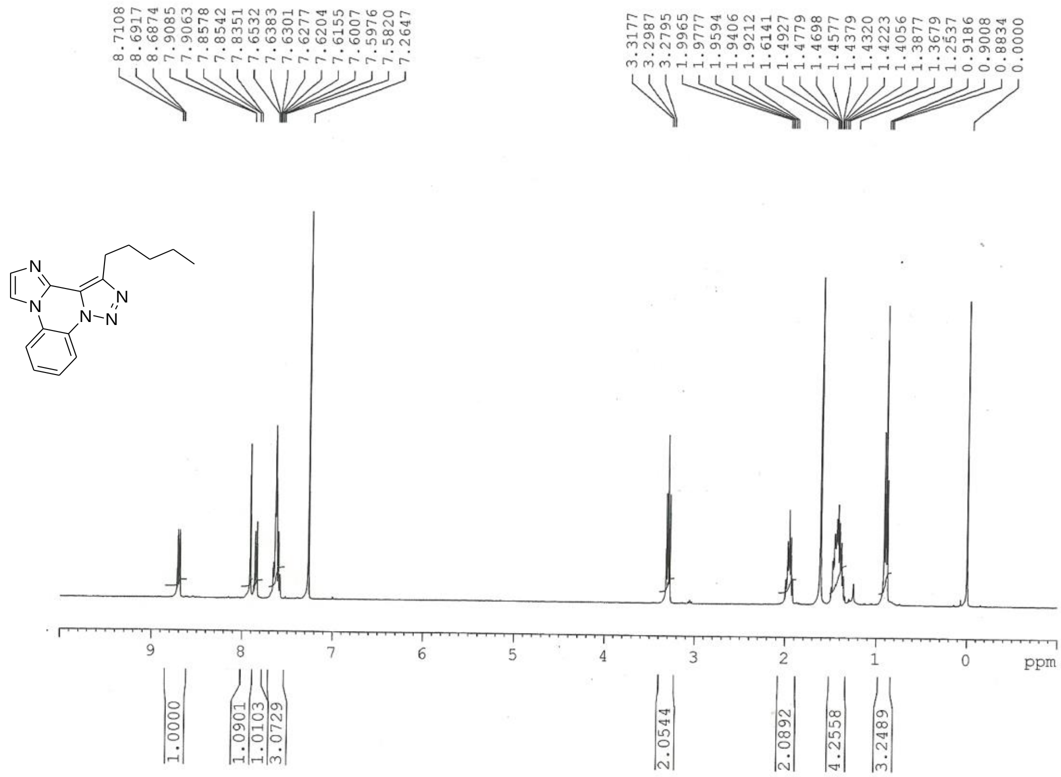

^{1}H NMR spectrum of **4k**

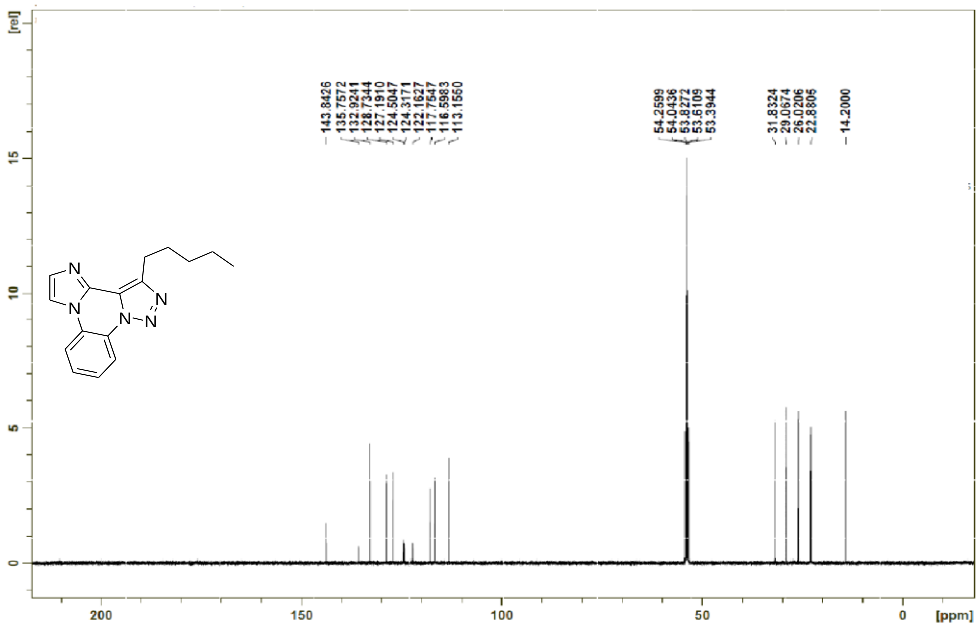

^{13}C NMR spectrum of **4k**

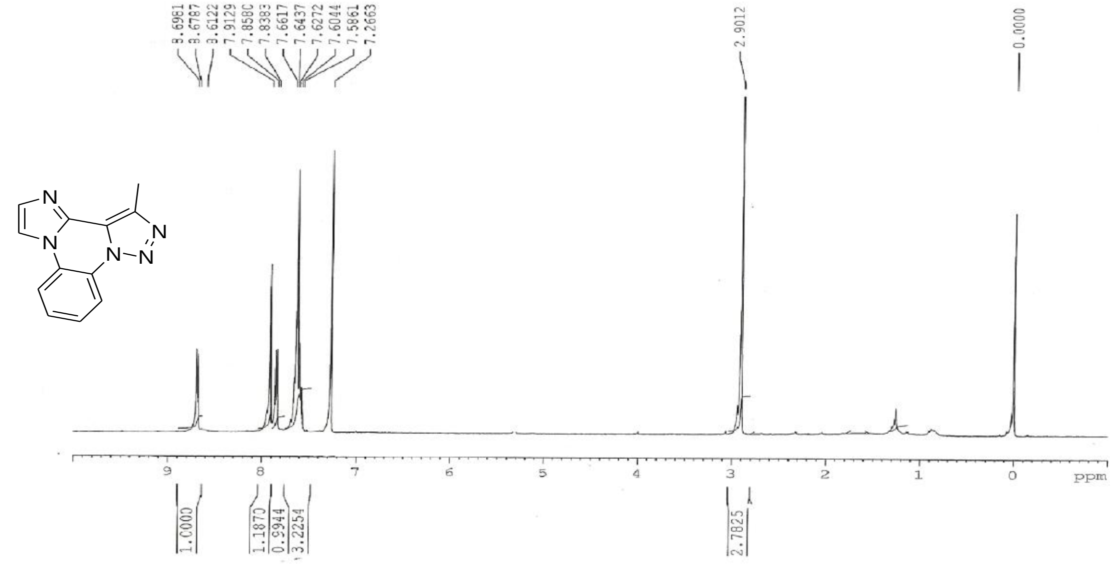

^{1}H NMR spectrum of **4l**

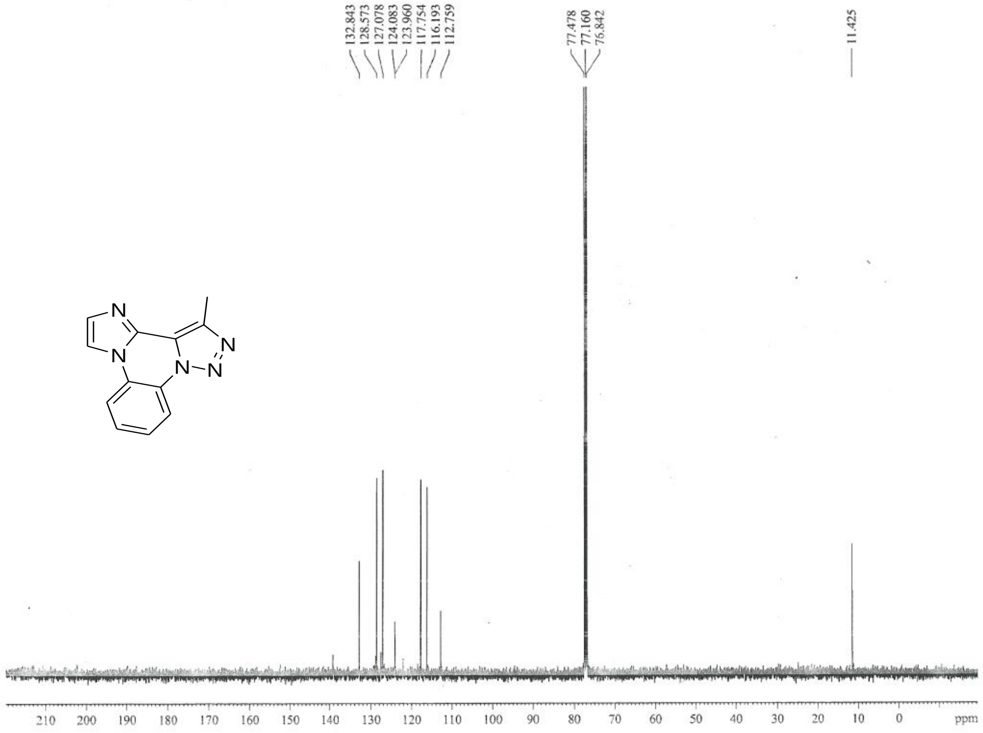

^{13}C NMR spectrum of **4l**

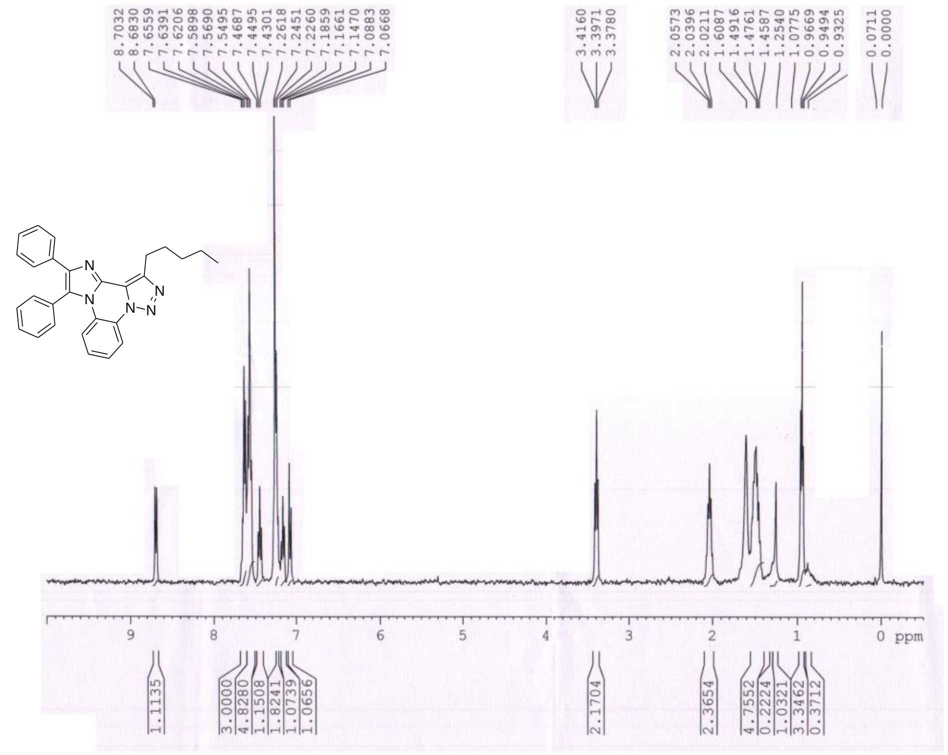

^{1}H NMR spectrum of **4m**

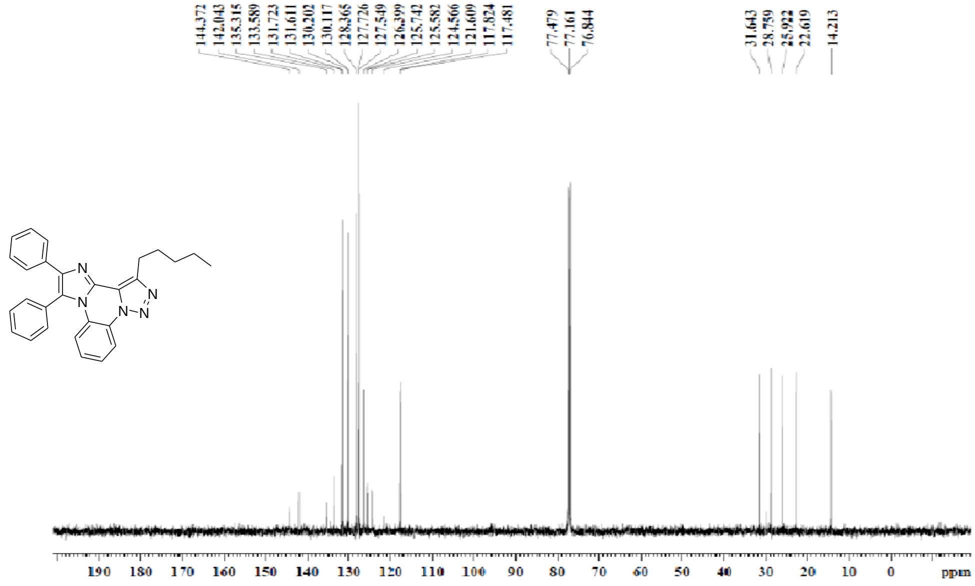

^{13}C NMR spectrum of **4m**
